# Available options for doing more with less: laboraory automation as one tool in the arsenal

**DOI:** 10.1155/S146392469800011X

**Published:** 1998

**Authors:** Stephen Scypinski, John Baiano, Theodore Sadlowski

**Affiliations:** 1 Pharmaceutical Research and Development Hoffmann-La Roche Inc. Nutley NJ USA; 2 R. W. Johnson Pharmaceutical Research Institute Po. Box 300 Route 202 Rorifan New Jersey 08869 USA

## Abstract

Projects that require analytical support can evolve from a number of different situations, for example new molecular entities from drug discovery; process changes; packaging changes; site changes; line extensions; and inlicensed projects and compounds. Laboratory automation has been shown to provide a viable and practical solution to assisting in analytical development. However, it is not always the most logical answer. A truly flexible and responsive analytical unit will make a decision on a case-by-case basis, when faced with a new project, whether it is best to: automate some or all aspects/testing involved; contract out to a reputable and approved contract research organization (CRO); hire temporary help; use available in-house resources; use a combination of the options shown above (for example to evaluate the complexity of the new project versus what the in-house resources are currently working on). The paper discusses the advantages and disadvantages of the various options with respect to providing analytical support and suggests optionsfor the most effective use of resources. The role of automation as one of the important tools in the arsenal of these options is highlighted.


Journal of Automatic Chemistry, Vol. 20, No. 3 (May–June 1998) pp. 87–89

### Available options for doing more with less: laboratory automation as one tool in the arsenal

**Stephen Scypinski,* John Baiano and Theodore Sadlowski**

*Pharmaceutical Research and Development, Hoffmann-La Roche Inc., Nutley, NJ, USA*

*Projects that require analytical support can evolve from a number of different situations, for example new molecular entities from drug discovery; process changes; packaging changes; site changes; line extensions; and inlicensed projects and compounds. Laboratory automation has been shown to provide a viable and practical solution to assisting in analytical development. However, it is not always the most logical answer. A truly flexible and responsive analytical unit will make a decision on a case-by-case basis, when faced with a new project, whether it is best to: automate some or all aspects/testing involved; contract out to a reputable and approved contract research organization (CRO); hire temporary help; use available in-house resources; use a combination of the options shown above (for example to evaluate the complexity of the new project versus what the in-house resources are currently working on). The paper discusses the advantages and disadvantages of the various options with respect to providing analytical support and suggests options for the most effective use of resources. The role of automation as one of the important tools in the arsenal of these options is highlighted.*

### The drug discovery and development process

Most are familiar with the general concept of drug discovery and development and it is not the focus of this particular paper to discuss the various stages of the development process in detail. However, in general, the life cycle of a drug product follows the stages below:

- *Pre-Phase 0 or 'Phase 00'.* This is actually not a stage of development but rather, the final stage in the discovery process. It involves screening of multiple candidates against one or more screens or *in vitro* assay models to determine biological activity of a potential drug candidate. The end of this phase is marked by the selection of a discrete candidate molecule (or very small number of molecular analogues) which are presented to management and proposed for further development.
- *Phase 0.* During Phase 0 the candidate molecule is readied for the start of the clinical trials. Toxicology studies are performed and the molecular forms are characterized. Development of a suitable formulation is also done during this time. If applicable, regulatory documentation, in the form of an investigational new drug (IND) file is prepared. The culmination of Phase 0 is marked by the dosing of the first clinical patient.
- *Phase I.* The beginning of the clinical programme marks not only the end of Phase 0, but also the beginning of Phase I. These early clinical trials seek to demonstrate the feasibility of the drug with respect to patient tolerance and the achievement of desired blood levels and other related pharmacokinetic parameters. If the drug is not excessively toxic or is not being used for such indications as cancer and AIDS, the study is done with healthy volunteers. Successful findings in the Phase I 'Proof-of-Concept' trial leads to a higher level trial, that of the Phase II dose fining or dose ranging studies.
- *Phase II.* The goals of the Phase II trials are to show a dose response for the drug and to explore adverse events, so that the highest efficacious dose may be given without causing undesirable effects. Phase II studies (and all clinical studies after Phase I) are done on actual patients. Such trials are supervised by medically qualified specialists. At some point in the latter stage of the Phase II trials, the company developing the drug will conduct a business analysis and make a financial commitment to continue developing the compound, for the Phase III trials will consume considerable resources and funds.
- *Phase III.* The largest and most costly stage of drug development is the multi-centre Phase III trials, which are usually conducted globally. The goal of Phase III is to demonstrate efficacy in a controlled population of patients. It is the data from Phase III that determines the final label information and ultimately how the product will be viewed by the public.
- *Pre-NDA Phase.* Even while the Phase III trials are continuing, the preparation for the filing of the new drug application (NDA) is already underway. The regulatory documentation is prepared and filed with the FDA (and other regulatory bodies if applicable) and the site is readied for the impending pre-approval inspection (PAI).
- *Pre- and post-approval.* In the months both before and after approval of the NDA, dosage form life cycle planning will take place, additional Phase IIIB and Phase IV clinical studies will begin, and technical commitments made to the regulatory authorities both during review of the application and site inspection will be dealt with. The development of line extensions and novel delivery systems, and new strengths of the active for new clinical indications

* Present address: R. W. Johnson Pharmaceutical Research Institute, Po. Box 300, Route 202, Rorifan, New Jersey 08869, USA.

0142-0453/98 $12·00 © 1998 Taylor & Francis Ltd are done to maximize return on investment and extend the lifetime of the product.

While the above discussion centres around the 'normal' course of drug discovery and development, starting within a lead discovery centre and transferring to a development organization, all of us in the technical or nonclinical areas of drug development know that the pipeline from drug discovery to development is only one of many channels feeding the river of drug development. In addition to the activities surrounding the bridging of a particular molecule from concept to reality to marketed product, the everyday work in a technical and for the rest of this article, analytical, environment can stem from other sources as well:

- Development work from other discovery or lead generation centres.
- Strategic alliances between the company and other scientific venues, leading to a partnership or codevelopment of the drug.
- Inlicensed molecular entities or more mature projects where the developing organization will buy the compound outright.
- New formulations and/or novel delivery systems for existing marketed products.
- Development of a generic introduction for a mature marketed product (if the developing company has a generic division).
- Transfer of a synthetic or dosage form manufacturing process to another site.
- Alternate packaging presentations for existing products.
- Problem-solving or analytical investigations surrounding either an existing marketed product or product under development.

What all of these sources have in common is that they generate a lot of work. While some of the work may be classified as routine, for example, stability samples for the registration of alternate package configurations or new formulations, there may be considerable amounts of method development, validation, troubleshooting, preparation and/or review of regulatory documentation, and formulation and process support.

### Options for handling the analytical workload

Analytical laboratory managers have become quite adept at handling additional projects within their portfolio of work and, at first glance to the customer, it may seem that they must have an excess of personnel to draw upon so that not even a single project or sample request suffers a missed deadline. Besides prioritizing analyses and being proactive in project planning, there are several options available to the analytical laboratory to handle these projects. The most prominent of these are:

- Automation of entire methods and/or operations. Examples here include the use of dissolution robots/workstations or tablet processing workstations to perform tasks which lead to the evaluation of large numbers of samples.
- Use of 'partial automation' as a means to move samples through a particular portion of a method that may be repetitious or time-consuming, or even to improve method precision in this operation. Such examples as pipetting, dispensing or diluting steps as portions of larger methods fall into this category.
- Employing available in-house resources to handle the project (if those resources are available).
- Reallocate resources from another project to the new project if its priority in the organization is higher.
- Hire temporary help to handle the peaks in the project timeline.
- Use a contract research organization (CRO) as an outsourcing partner for either a portion of the project or the entire project.

Realizing that there are always options to getting the job done and keeping the needs of the customer in mind, the needs and demands of the individual projects in the work portfolio and the degree of complexity and manpower required to fulfil commitments need to be considered. Examining the options available, the next set of questions that needs to be addressed is the following:

- what is the timeframe of the new project relative to the other projects already underway in the organization?
- What is the nature of the new work? Is it routine or demanding? Will extensive training be required for the personnel who are slated to perform the work?
- Who are the partners, collaborators and customers in the work? Who is the project driver?
- What is the level of cGMP compliance required in the project? Will the resulting data be a part of a regulatory submission?
- Is automation a realistic option? Will considerable lead time be required to develop and validate an automated method? Will the resulting number of samples processed justify the up-front resources needed to integrate automation into the project?
- Is cost an issue of concern?

In trying to make decisions as to what the best course of action would be, the analytical manager needs to consider all options, both for the new project and those already in his/her laboratory and the best use of the resources and money available to get the job done. For example, having every experienced, advanced level analytical scientists perform routine stability analyses is a poor use of talent when these chemists could be applying their skills toward such tasks as method development, validation and problem solving. Such analytical testing would be best performed by temporary personnel. Conversely, the option to place such studies at a CRO is another option if the timeframe allows for the working up of a quotation with the company and the execution of an analytical transfer protocol. If the CRO has not been used before, an audit of the facility by the compliance or quality assurance group will have to be done as well. Contracting is becoming quite popular in the pharmaceutical industry today and the number of CROs in increasing at a rapid rate. Indeed, many research-based pharmaceutical companies are developing contractual arrangements in the form of strategic alliances with the CROs to the extent that the CRO is considered a partner in the development cycle. Examples of this include entrusting the entire registration stability program for a series of molecules to a reputable CRO or allowing a CRO to actually drive the development of a new molecular entity (NME) to the extent where the contractor would perform such early development work as preformulation and excipient compatibility studies, analytical method development, screening and development of a Phase I dosage form, toxicology studies and toxicokinetic analysis. As the competition between corporation becomes even more fierce, it is expected that the use of CROs will increase even further in the coming years. One of the key advantages of outsourcing is that it allows in-house resources to be devoted to other important projects, possibly those where quick turn-around times are needed or where the customer is in close proximity. It also allows the resident staff to pursue scientifically challenging projects and/or pursue process improvement ideas and technologies, examples which may include new analytical techniques and instrumentation. The drawbacks to outsourcing are:

- Even simple studies cost considerable money.
- Methods transfer must be considered (the time and money).
- Communications may become an issue if not properly managed. This also depends on the geographical location of the CRO relative to the company.
- The involvement of a quality assurance organization to monitor the cGMP compliance level of the CRO is required.
- If the project is developmental, such that it will lead to clinical studies and eventually to a regulatory filing, the CRO will become an additional site for a Pre-Approval Inspection (PAI). It is the absolute responsibility of the hiring company to make sure the CRO is compliant and prepared for the inspection when it occurs.
- Outsourcing still requires in-house resources in the form of personnel to manage the data coming from the CRO and handle the billing and invoicing that accompanies the data.
- If the project contracted out is one which will result in a regulatory filing, not performing the work in-house results in a loss of expertise in that the experience with the NME, formulation and project is not fostered in-house, but rather at the CRO. This may not be devastating, although turnover at most CROs has been an issue of concern with hiring companies.

As an alternative to the use of CROs, temporary employees can be hired to handle additional work. The advantages of using such workers is that they consume very little overhead expenses (i.e. benefits), their management is done through contract, they offer a pool of probationary workers which can be drawn upon if and when positions become available. One disadvantage (especially at this point in time) is that the upswing in the pharmaceutical industry job market is making good temporary analysts hard to keep unless they are made permanent. Another drawback which ties into the previous point is that the ownership of the project is not fostered with the permanent scientific staff of the company and if the temporary employee resigns, then the knowledge and project-related experience will depart with that person. Some temporary workers make looking for a permanent position a priority, which can be problematic, especially in larger organization.

Last but not least, is the use of robotics and automation in an environment of limited resources. Indeed, there have been a plethora of presentations at the ISLAR meetings over the past several years lauding the virtues of automation for a variety of situations and tasks. Caution should be exercised that the 'correct' matches for the abilities of robotics and the demands of the project be established. One must also remember the validation criteria for a robotic system that will be used in a cGMP application. Training of the personnel who will use the robot for analysis must also be considered. The analysis of the resulting data must be a priority of using the robot in a timesaving mode will be for naught. It should be pointed out that utilizing laboratory robotics may cause slight changes in the local areas in which they are being utilized. For example, stability setup for a particular project, most often consisting of multiple lots and/or package configurations, are set up in such a fashion so as to be staggered with respect to their analysis dates so the analytical laboratory is not overwhelmed with samples followed by a complete lapse in samples. When using a robot to perform the same analyses, it is more efficient to cause all the samples to come off station simultaneously so the robot is utilized in its night and day analytical mode. This also saves the operators the chore of running standards for the robot multiple times.

### Conclusions

- Laboratory automation offers but one solution to the ever expanding analytical work portfolio.
- Robotics are not the answer to the needs of every project.
- One needs to consider both the type and volume of work, as well as the priority, when choosing a solution to a problem.
- Both outsourcing and temporary employees have advantages and disadvantages when used to offset increased workload.

